# Supplementary material for: Prevalence of diabetes and associated risk factors in Ga Mashie, Accra, Ghana: a cross-sectional CARE Diabetes community-based survey
Source: BMJ Open. 2026 May 3;16(4):e119125. doi: 10.1136/bmjopen-2026-119125 (PMC13140948; doi:10.1136/bmjopen-2026-119125)
Supplement: online supplemental file 1 [file bmjopen-16-4-s001.docx]

# **SUPPLEMENTARY MATERIALS.**

**Table S1.** Age, wealth, and sex of eligible and surveyed household members.

|  | Eligible | | Surveyed | | Non-surveyed^*^ |
| --- | --- | --- | --- | --- | --- |
| Sex | N | % (95% CI) | n | % (95% CI) | n/N (%) |
| Male | 381 | 38.7 (36.0; 41.6) | 305 | 36.3 (33.2; 39.6) | 76/381 (19.9%) |
| Female | 626 | 61.3 (58.5; 64.0) | 549 | 63.7 (60.4; 66.8) | 77/626 (12.3%) |
| Wealth tertile | n | % (95% CI) | n | % (95% CI) | n (%) |
| Most poor | 315 | 30.6 (24.9; 37.0) | 277 | 33.0 (27.9; 38.5) | 30/315 (12.1%) |
| Poor | 340 | 33.0 (27.7; 38.9) | 284 | 32.3 (27.3; 37.8) | 56/340 (16.5%) |
| Least Poor | 352 | 36.4 (31.1; 42.0) | 293 | 34.7 (29.6; 40.2) | 59/352 (16.8%) |
| Age category | n | % (95% CI) | n | % (95% CI) | n (%) |
| 25-44 years | 499 | 49.2 (45.7; 52.7) | 397 | 46.2 (42.3; 50.1) | 102/499 (20.4%) |
| 45-64 years | 374 | 37.6 (34.6; 40.6) | 328 | 38.6 (35.2; 42.1) | 46/374 (12.3%) |
| ≥65 years | 134 | 13.3 (10.9; 16.0) | 129 | 15.3 (13.0; 17.9) | 5/134 (3.73%) |
| Total | 1,007 | 100% | 854 | 100% | 153/1,007 (15.2%) |

^*^ The percentage of non-surveyed household members is in relation to the eligible sample.

**Table S2.** Household characteristics.

|  |  |  | Wealth Tertiles | | | | | |
| --- | --- | --- | --- | --- | --- | --- | --- | --- |
|  | Total (n=644) | | Most poor (n=215) | | Poor (n=215) | | Least poor (n=214) | |
| Characteristic | % or mean | 95% CI | % or mean | 95% CI | % or mean | 95% CI | % or mean | 95% CI |
| Household size (members) | 2.84 | (2.76, 2.92) | 2.51 | (2.41, 2.61) | 2.92 | (2.79, 3.05) | 3.06 | (2.93, 3.19) |
| Female headed household (%) | 52.9 | (48.7, 57.0) | ***64.7*** | ***(58.6, 70.4)*** | ***54.0*** | ***(46.4, 61.4)*** | ***40.0*** | ***(32.6, 47.9)*** |
| Female member (%) | 57.9 | (55.3, 60.4) | 58.9 | (54.8, 62.8) | 59.8 | (55.5, 64.0) | 55.3 | (50.7, 59.9) |
| Family structure (%) |  |  |  |  |  |  |  |  |
| child dependent (<15 years) | 23.4 | (21.0, 26.1) | ***17.5*** | ***(13.0, 23.3)*** | ***25.2*** | ***(22.0, 28.7)*** | ***26.4*** | ***(22.1, 31.3)*** |
| working age adult (15-64 years) | 69.2 | (66.8, 71.6) | ***72.0*** | ***(66.6, 76.8)*** | ***66.8*** | ***(63.1, 70.3)*** | ***69.3*** | ***(64.5, 73.7)*** |
| aged dependent (≥65 years) | 7.30 | (5.9, 9.0) | ***10.5*** | ***(7.8, 13.9)*** | ***8.00*** | ***(6.0, 10.5)*** | ***4.30*** | ***(2.6, 6.8)*** |
| Household with a pregnant woman (%) | 5.70 | (4.4, 7.4) | ***8.1*** | ***(5.4, 12.0)*** | ***2.6*** | ***(1.3, 4.9)*** | ***6.7*** | ***(4.6, 9.7)*** |
| Shared toilet with other households (%) | 89.6 | (86.4, 92.2) | 94.3 | (89.6, 96.9) | 92.7 | (87.1, 95.9) | 83.1 | (75.5, 88.7) |
| Fuel for Cooking (%) |  |  |  |  |  |  |  |  |
| Charcoal | 65.7 | (61.4, 69.8) | ***86.9*** | ***(82.1, 90.6)*** | ***74.0*** | ***(66.3, 80.5)*** | ***37.5*** | ***(29.9, 45.8)*** |
| LPG | 29.6 | (25.8, 33.7) | ***8.6*** | ***(5.4, 13.5)*** | ***21.1*** | ***(15.1, 28.5)*** | ***57.8*** | ***(49.6, 65.6)*** |

We used chi-square to test for associations. Bold numbers represent a significant association (p<0.05).
