# Supplementary material for: Prevalence of diabetes and associated risk factors in Ga Mashie, Accra, Ghana: a cross-sectional CARE Diabetes community-based survey
Source: BMJ Open. 2026 May 3;16(4):e119125. doi: 10.1136/bmjopen-2026-119125 (PMC13140948; doi:10.1136/bmjopen-2026-119125)
Supplement: Supplementary file 2 [file bmjopen-2026-119125.reviewer_comments.pdf]

PEER REVIEW HISTORY

BMJ Open publishes all reviews undertaken for accepted manuscripts. Reviewers are asked to complete a checklist review form and are provided with free text boxes to elaborate on their assessment. These free text comments are reproduced below.

ARTICLE DETAILS

Title (Provisional)

Prevalence of diabetes and associated risk factors in Ga Mashie, Accra, Ghana – a cross-sectional CARE Diabetes community-based survey.

Authors

Grijalva-Eternod, Carlos; Sedzro, Kojo Mensah; Adjaye-Gbewonyo, Kafui; Kushitor , Sandra Boatemaa; Lule, Swaib; Kushitor, Mawuli K; Marphatia, Akanksha A; Gray, Ethan; Amon, Samuel; Sanuade, Olutobi Adekunle; Awuah, Raphael; Baatiema, Leonard; Kretchy, Irene A.; Arhinful, Daniel; Koram, Kwadwo K.; Fottrell, Edward

---

VERSION 1 - REVIEW

**Reviewer**	**1**
**Name**	**Adrian Heald**
**Affiliation**	**Salford Royal Hospitals NHS Trust, The University of Manchester**
**Date**	**11-Mar-2025**
**COI**	**None**

Comments to the Author:
This is an excellent paper which is testament to the huge amount of work that has gone into the study undertaken in Ghana

Some minor points only
Page 4 around line 21 – this should read BMI≥30 not >30

Methods:
Did the research team go back at least once to a household if there was no one at home first time round?

How often was the glucose meter checked against lab glucose and / or calibrated?

Discussion
Please can the authors mention 2 relevant papers

1. Concerning the higher diabetes prevalence in women – a relevant UK based paper looking at a predominant Caucasian population reported similar findings in 2023. Please can this be cited if the authors agree - Stedman M, Whyte MB, Laing I, Fryer AA, Torres BM, Robinson A, Mannan F, Gibson JM, Rayman G, Heald AH. Failure to control conventional cardiovascular risk factors in women with type 2

diabetes might explain worse mortality. Diabetes Metab Res Rev. 2023 Nov;39(8):e3695

2. Concerning the potential influence of carbohydrate intake on rates of diabetes, a paper looked at macronutrient intake in representative samples and found that a higher carbohydrate intake relative to fat and protein associated with lower rates of diabetes in Africans living in West Africa than the higher relative consumption of fat and protein in African origin individuals living in the Caribbean and Europe who had higher rates of diabetes. The study showed independent effects of dietary factors on hyperglycaemia in African origin populations. Please can this be cited if the authors agree -
Anderson SG, Younger N, Heald AH, Tulloch-Reid MK, Simukonda WP, Mbanya JC, Jackson MD, Balkau B, Sharma S, Tanya A, Forrester T, Wilks R, Cruickshank JK. Nutrient intakes and dysglycaemia in populations of West African origin. Br J Nutr. 2011 Jan;105(2):297-306

**Reviewer**	**2**
**Name**	**Fredirick Mashili**
**Affiliation**	**Muhimbili University of Health and Allied Sciences**
**Date**	**11-Mar-2025**
**COI**	**None**

Comments to the Author:
The authors aimed to establish the prevalence of diabetes and associated risk factors in Mashie, Accra, Ghana using a cross sectional study design. They report a prevalence of 8.2%, with notable social demographic disparities in the distribution of diabetes and associated risk factors. These findings are important given the raising burden of diabetes in the region. The study was meticulously designed, carried out and analyzed. Additionally, the manuscript is very well written.

Despite these strengths, few major and minor revision will improve the quality of this manuscript.

Major concerns
1. The authors used random blood glucose test to diagnose diabetes and found a higher prevalence of the disease than previously reported in Ghana. Based on that a conclusion that diabetes is increasing in Ghana may be reached. While this can be true, the use of RBG which can easily be affected by food eaten prior to the test could have resulted in overestimation of the true prevalence. It should be noted that guidelines that allow the use of RBG in diagnosing diabetes recommend two subsequent RBG tests for diabetes to be confirmed. Or RBG has to be complimented by typical symptoms of diabetes. Given difficulties in repeating measurements in a research setting, why didn't the authors consider the inclusion of symptom questions in their questionnaire to compliment their RBG use? While they mentioned the use of RBG as a limitation, the discussion to justify its use given the possibility of overestimating prevalence is insufficient. More introduction and discussion to justify the use of RBG despite the known shortcomings is important.
https://jamanetwork.com/journals/jamainternalmedicine/fullarticle/412871,
https://www.aafp.org/pubs/afp/issues/2016/0115/p103.html,
https://pmc.ncbi.nlm.nih.gov/articles/PMC4399288/,
https://pmc.ncbi.nlm.nih.gov/articles/PMC4399288/

One notable finding is gender differences in prevalence of diabetes, obesity and physical activity level. Women who were found to have significantly higher prevalence of diabetes, also had higher prevalence of obesity and physical inactivity. They were also found to have relatively unfavorable socioeconomic status. Social economic status was somehow linked to physical inactivity. Applying critical thinking looks like those factors modify each other. It could possibly be that women are more likely to get diabetes because they are more likely to be inactive and become obese. In situations like these exploring relationships/associations between variables become very important to an extent of being able to identify one independent variable. Why did the authors only used unadjusted models as they clearly stated that in the abstract. Both bivariate and multivariate analysis seems very important in this case

given the possibility of variables interference. They should therefore perform these analysis or clearly justify why they did not do that.

Minor comments
Since the title focuses on diabetes, line 28 (abstract, results) should just say diabetes and not non communicable disease risk factors. That can be mentioned in the discussion as appearing on the abstract is confusing as to whether the study focused on diabetes or NCDs.

Line 23 (introduction) "diabetes prevalence in..........should say where to avoid confusion of where that prevalence is refereeing to.

VERSION 1 - AUTHOR RESPONSE

Reviewer: 1
Dr. Adrian Heald, Salford Royal Hospitals NHS Trust, The University of Manchester
Comments to the Author:
This is an excellent paper which is testament to the huge amount of work that has gone into the study undertaken in Ghana

We thank the reviewer for their comments.

Some minor points only
Page 4 around line 21 – this should read BMI^{3}30 not >30

We have changed this to ≥30 where applicable.

Methods:
Did the research team go back at least once to a household if there was no one at home first time round?

We made up to three attempts to contact the household and obtain consent for participation in our study. We have added this detail to the Methods section.

How often was the glucose meter checked against lab glucose and / or calibrated?
We checked the glucometer against a reference standard on every use by comparing the calibration number displayed upon insertion of the test strip with the reference value printed on the strip box. We have added this detail to the Methods section.

Discussion
Please can the authors mention 2 relevant papers

1. Concerning the higher diabetes prevalence in women – a relevant UK based paper

looking at a predominant Caucasian population reported similar findings in 2023. Please can this be cited if the authors agree - Stedman M, Whyte MB, Laing I, Fryer AA, Torres BM, Robinson A, Mannan F, Gibson JM, Rayman G, Heald AH. Failure to control conventional cardiovascular risk factors in women with type 2 diabetes might explain worse mortality. Diabetes Metab Res Rev. 2023 Nov;39(8):e3695

We thank the reviewer for this suggestion. However, we feel the study population in this paper is of limited relevance to our study context in Ghana.

2. Concerning the potential influence of carbohydrate intake on rates of diabetes, a paper looked at macronutrient intake in representative samples and found that a higher carbohydrate intake relative to fat and protein associated with lower rates of diabetes in Africans living in West Africa than the higher relative consumption of fat and protein in African origin individuals living in the Caribbean and Europe who had higher rates of diabetes. The study showed independent effects of dietary factors on hyperglycaemia in African origin populations. Please can this be cited if the authors agree -
Anderson SG, Younger N, Heald AH, Tulloch-Reid MK, Simukonda WP, Mbanya JC, Jackson MD, Balkau B, Sharma S, Tanya A, Forrester T, Wilks R, Cruickshank JK. Nutrient intakes and dysglycaemia in populations of West African origin. Br J Nutr. 2011 Jan;105(2):297-306

We thank the reviewer for alerting us to this relevant evidence, which supports our discussion on understanding diet in different contexts. We have now cited this paper in our manuscript.

Reviewer: 2
Dr. Fredirick Mashili, Muhimbili University of Health and Allied Sciences
Comments to the Author:
The authors aimed to establish the prevalence of diabetes and associated risk factors in Mashie, Accra, Ghana using a cross sectional study design. They report a prevalence of 8.2%, with notable social demographic disparities in the distribution of diabetes and associated risk factors. These findings are important given the raising burden of diabetes in the region. The study was meticulously designed, carried out and analyzed. Additionally, the manuscript is very well written.

Despite these strengths, few major and minor revision will improve the quality of this manuscript.

We thank Dr Mashili for their appraisal of our work and for their constructive review.

Major concerns

1. The authors used random blood glucose test to diagnose diabetes and found a higher prevalence of the disease than previously reported in Ghana. Based on that a conclusion that diabetes is increasing in Ghana may be reached. While this can be true, the use of RBG which can easily be affected by food eaten prior to the test could have resulted in overestimation of the true prevalence. It should be noted that guidelines that allow the use of RBG in diagnosing diabetes recommend two subsequent RBG tests for diabetes to be confirmed. Or RBG has to be complimented by typical symptoms of diabetes. Given difficulties in repeating measurements in a research setting, why didn't the authors consider the inclusion of symptom questions in their questionnaire to compliment their RBG use? While they mentioned the use of RBG as a limitation, the discussion to justify its use given the possibility of overestimating prevalence is insufficient. More introduction and discussion to justify the use of RBG despite the known shortcomings is important. https://jamanetwork.com/journals/jamainternalmedicine/fullarticle/412871, https://www.aafp.org/pubs/afp/issues/2016/0115/p103.html, https://pmc.ncbi.nlm.nih.gov/articles/PMC4399288/, https://pmc.ncbi.nlm.nih.gov/articles/PMC4399288/

As described in our paper, RBG was the only feasible option for our epidemiological study. We have taken care to describe its limitations and to emphasise that this does not constitute a clinical diagnostic test, but is an acceptable method in epidemiological studies. We have now expanded our discussion of the limitations of RBG and acknowledge that it may overestimate the risk of diabetes.

We did not include self-reported symptom questions because we felt that the ability to reliably recognise, recall, and report valid symptoms of diabetes would potentially be influenced by education, health literacy, and prior experience with medical conditions and health services in our study context. Given the need to keep the interview tool concise, we did not consider the inclusion of such questions to be particularly useful or reliable in this setting.

One notable finding is gender differences in prevalence of diabetes, obesity and physical activity level. Women who were found to have significantly higher prevalence of diabetes, also had higher prevalence of obesity and physical inactivity. They were also found to have relatively unfavorable socioeconomic status. Social economic status was somehow linked to physical inactivity. Applying critical thinking looks like those factors modify each other. It could possibly be that women are more likely to get diabetes because they are more likely to be inactive and become obese. In situations like these exploring relationships/associations between variables become very important to an extent of being able to identify one independent variable. Why did the authors only used unadjusted models as they clearly stated that in the abstract. Both bivariate and multivariate analysis seems very important in this case given the possibility of variables

interference. They should therefore perform these analysis or clearly justify why they did not do that.

We agree with the reviewer that there is likely to be a complex interplay between the physical, social, and behavioural parameters measured in our study. The cross-sectional nature of our survey limits our ability to disentangle causal relationships, and more complex analytical procedures are beyond the scope of this initial descriptive paper. Our presentation of unadjusted data aligns with the aims of descriptive epidemiology and serves as an initial description of diabetes and NCD risk in the study area.

Analysis involving adjustments, as suggested by the reviewer, require either established conceptual presentation frameworks or prior evidence to justify the treatment of specific parameters as confounders or effect modifiers. These are the focus of ongoing analyses within the broader CARE Diabetes study. We have now expanded the justification for our analytical approach and acknowledge its limitations in the revised manuscript.

Minor comments
Since the title focuses on diabetes, line 28 (abstract, results) should just say diabetes and not non communicable disease risk factors. That can be mentioned in the discussion as appearing on the abstract is confusing as to whether the study focused on diabetes or NCDs.

While recognising the overlap between key risk factors and leading NCDs, we have edited the Results section of the Abstract to avoid any misunderstanding regarding the diabetes focus of our paper.

Line 23 (introduction) "diabetes prevalence in..........should say where to avoid confusion of where that prevalence is refereeing to.

We have clarified in the text that this refers to global diabetes prevalence.

Reviewer: 1
If you have selected ‘Yes’ above, please provide details of any competing interests.: NA

Reviewer: 2
If you have selected ‘Yes’ above, please provide details of any competing interests.: Not Applicable
